# Modulation of the Unfolded Protein Response by Tauroursodeoxycholic Acid Counteracts Apoptotic Cell Death and Fibrosis in a Mouse Model for Secondary Biliary Liver Fibrosis

**DOI:** 10.3390/ijms18010214

**Published:** 2017-01-20

**Authors:** Annelies Paridaens, Sarah Raevens, Lindsey Devisscher, Eliene Bogaerts, Xavier Verhelst, Anne Hoorens, Hans van Vlierberghe, Leo A. Van Grunsven, Anja Geerts, Isabelle Colle

**Affiliations:** 1 Department of Hepatology and Gastroenterology, Ghent University, 9000 Ghent, Belgium; Annelies.paridaens@ugent.be (A.P.); Sarah.Raevens@ugent.be (S.R.); Lindsey.Devisscher@ugent.be (L.D.); Eliene.Bogaerts@ugent.be (E.B.); Xavier.Verhelst@ugent.be (X.V.); Hans.Vanvlierberghe@ugent.be (H.V.V.); Isabelle.Colle@ugent.be (I.C.); 2 Department of Pathology, Ghent University, 9000 Ghent, Belgium; Anne.hoorens@ugent.be; 3 Liver Cell Biology Lab, Vrije Universiteit Brussel, 1090 Brussels, Belgium; lvgrunsv@vub.ac.be

**Keywords:** liver fibrosis, cirrhosis, tauroursodeoxycholic acid, endoplasmic reticulum stress, unfolded protein response, apoptosis, cell death

## Abstract

The role of endoplasmic reticulum stress and the unfolded protein response (UPR) in cholestatic liver disease and fibrosis is not fully unraveled. Tauroursodeoxycholic acid (TUDCA), a hydrophilic bile acid, has been shown to reduce endoplasmic reticulum (ER) stress and counteract apoptosis in different pathologies. We aimed to investigate the therapeutic potential of TUDCA in experimental secondary biliary liver fibrosis in mice, induced by common bile duct ligation. The kinetics of the hepatic UPR and apoptosis during the development of biliary fibrosis was studied by measuring markers at six different timepoints post-surgery by qPCR and Western blot. Next, we investigated the therapeutic potential of TUDCA, 10 mg/kg/day in drinking water, on liver damage (AST/ALT levels) and fibrosis (Sirius red-staining), in both a preventive and therapeutic setting. Common bile duct ligation resulted in the increased protein expression of CCAAT/enhancer-binding protein homologous protein (CHOP) at all timepoints, along with upregulation of pro-apoptotic caspase 3 and 12, tumor necrosis factor receptor superfamily, member 1A (TNFRsf1a) and Fas-Associated protein with Death Domain (FADD) expression. Treatment with TUDCA led to a significant reduction of liver fibrosis, accompanied by a slight reduction of liver damage, decreased hepatic protein expression of CHOP and reduced gene and protein expression of pro-apoptotic markers. These data indicate that TUDCA exerts a beneficial effect on liver fibrosis in a model of cholestatic liver disease, and suggest that this effect might, at least in part, be attributed to decreased hepatic UPR signaling and apoptotic cell death.

## 1. Introduction

Cholestatic liver disease includes a spectrum of hepatobiliary pathologies and is characterized by impaired hepatobiliary production or excretion of bile, which causes biliary stasis and retention of bile acids [1]. Chronic cholestasis, caused by genetic defects, toxins, mechanical aberrations or dysregulation of the immune system, leads to increased bile acid accumulation, which induces liver damage and cell death via diverse mechanisms [2]. Hydrophobic bile acids are cytotoxic due to their detergent action on lipid components and potential to induce oxidative stress and mitochondrial dysfunction which eventually results in hepatocyte and cholangiocyte death [3]. This process, along with apoptotic debris and paracrine stimulation by neighboring cells, among many inducing factors, causes hepatic stellate cell activation, initiating progressive fibrosis [4,5].

Accumulation of bile acids is known to induce stress to the endoplasmic reticulum (ER) [6,7,8,9]. ER stress refers to a state triggered by a disruption of ER homeostasis, which results in the accumulation of unfolded proteins within the ER lumen [10]. There are three transmembrane ER stress sensors: the inositol-requiring enzyme 1 (IRE1), the activating transcription factor 6 (ATF6) and the PKR-like endoplasmic reticulum kinase (PERK). In normal conditions, these ER stress receptors are maintained in an inactive state through association with the ER chaperone glucose-regulated protein, 78 kDa (Grp78 or BiP). During ER stress, the accumulation of unfolded proteins triggers the dissociation of BiP, releasing the sensors and activating the IRE1, ATF6 and PERK pathways. This signaling is referred to as the unfolded protein response (UPR). The UPR functions as an adaptive mechanism, aiming to restore cellular homeostasis. However, if ER stress sustains, the UPR switches to ER stress-induced cell death signaling, which is characterized by the involvement of the pro-apoptotic transcription factor CCAAT/enhancer-binding protein homologous protein (CHOP) [11]. CHOP-mediated apoptotic cell death has been reported in both in vitro and in vivo experiments, as well as in acute and chronic liver pathologies such as acetaminophen overdose, non-alcoholic steatohepatitis and cholestasis [6,12,13,14]. However, the therapeutic potential to inhibit UPR-mediated liver damage following cholestasis in the evolution towards chronic liver disease and fibrosis has not been fully explored. In this context, we were interested in the hydrophilic bile acid tauroursodeoxycholic acid (TUDCA), which is known for its ER stress-reducing and anti-apoptotic capacities [15,16,17,18,19,20,21,22,23,24,25,26,27].

Tauroursodeoxycholic acid is the taurine-conjugated form of ursodeoxycholic acid (UDCA), which is FDA approved for the treatment of cholestatic liver disease, including primary biliary cirrhosis, and has no major adverse effects [28]. UDCA’s effectiveness is limited by its poor bioavailability, which can be improved by taurine conjugation. It has been demonstrated that TUDCA binds to the hydrophobic regions of proteins, thereby preventing protein aggregation and reducing ER stress and related apoptosis [29]. In addition, recently, the activation of CHOP was shown to stimulate the transcription of pro-caspase 1 and 11, which become activated upon cleavage and activation by NLRP3, the most important inflammasome [14].

In this study, we explored the hepatic UPR and cell death signature in time during the initiation and progression of cholestasis-induced liver fibrosis in mice, and investigated whether modulating these processes with TUDCA has a beneficial effect on liver damage and fibrosis. We found that the UPR response is activated after common bile duct ligation (CBDL) and that preventive and therapeutic treatment with TUDCA results in a significant reduction of liver fibrosis, which was associated with decreased expression of UPR and pro-apoptotic markers.

## 2. Results

### 2.1. Common Bile Duct Ligation Activates the Hepatic Unfolded Protein Response

**Figure 1:**
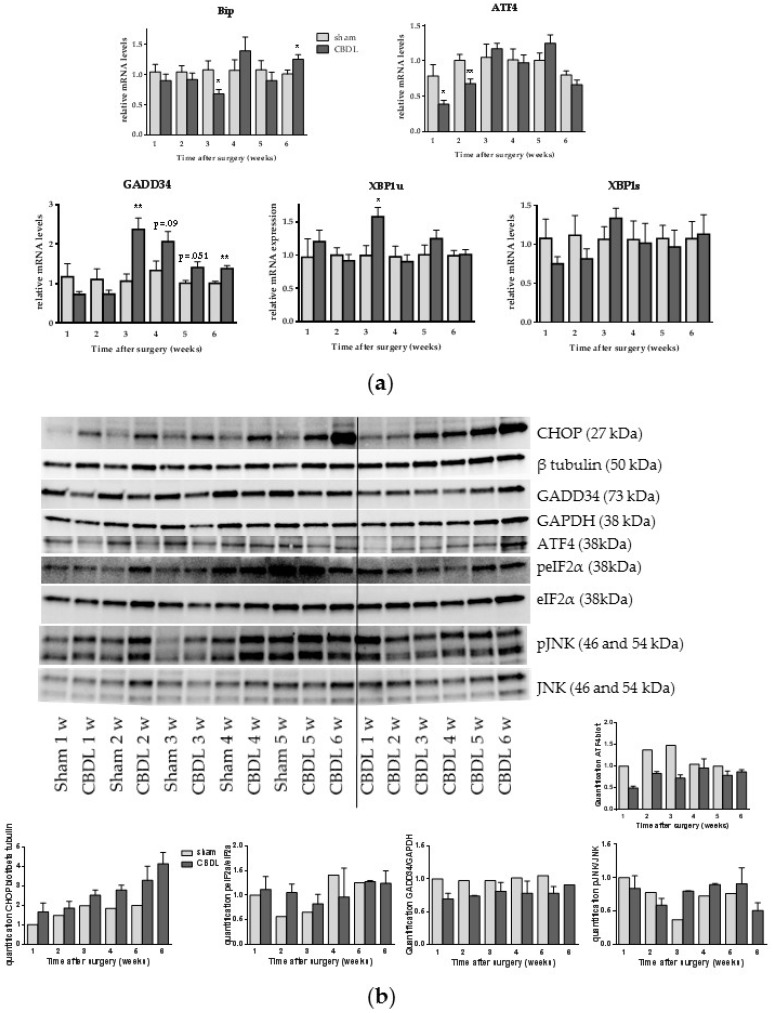
Common bile duct ligation (CBDL) activates the unfolded protein response. (**a**) Real-time quantitative polymerase chain reaction analysis of growth arrest and DNA damage-inducible protein (GADD34), unspliced X-box binding protein 1 (XBP1u) and spliced XBP1 (XBP1s); (**b**) Western blot and quantification of CCAAT/enhancer-binding protein homologous protein (CHOP), GADD34, activating transcription factor 4 (ATF4), (phosphorylated) eukaryotic initiation factor 2 alpha (p)eIF2α, and c-Jun N-terminal kinase (p)JNK on whole liver lysates. Results are representative of all samples: samples of three to five mice were mixed for blotting and two different pools of the CBDL samples were blotted. β-Tubulin and glyceraldehyde 3-phosphate dehydrogenase (GAPDH) were used for normalization. Data are presented as mean ± SD. * *p* < 0.05, ** *p* < 0.01.

We first investigated the UPR signature during the onset and progression of CBDL-induced liver fibrosis by analyzing mRNA or protein expression of different UPR markers. Mice were sacrificed on a weekly basis, starting from week 1 until week 6 after surgical induction. The mRNA levels of BiP were increased 6 weeks post CBDL. Activating transcription factor 4 (ATF4) and spliced X-box binding protein 1 (XBP1s) mRNA transcripts were not increased at any point following CBDL. Unspliced XBP1 (XBP1u) levels showed an increase at week 3 and growth arrest and DNA damage-inducible protein (GADD34) mRNA levels were significantly upregulated from week 3 until week 6 post-CBDL (Figure 1a). No increase in GADD34, ATF4 or phosphorylated eukaryotic initiation factor 2 α (peIF2α) protein levels could be detected, however, a gradual increase of CHOP expression in CBDL mice was seen on Western blot analysis starting at week 1 onwards to week 6 (Figure 1b). While c-Jun N-terminal kinase (JNK) activation is associated with apoptosis, we could not detect an increase in JNK phosphorylation in the time after CBDL (Figure 1b).

### 2.2. CBDL Induces a Pro-Apoptotic Response

**Figure 2:**
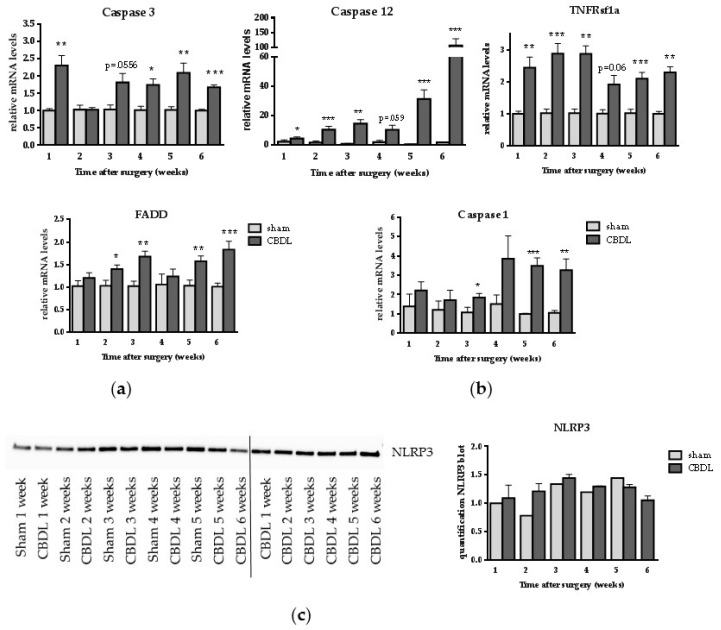
CBDL induces a pro-apoptotic response. (**a**) Real-time qPCR analysis of caspase 3, caspase 12, tumor necrosis factor receptor superfamily, member 1A (TNFRsf1a) and Fas-Associated protein with Death Domain (FADD); (**b**) Real time qPCR analysis of caspase 1; (**c**) Western blot of nucleotide binding, leucine-rich repeat and pyrin domains-containing protein 3 (NLRP3) on whole liver lysates. Western blot results are representative of all samples: samples of three to five mice were mixed for blotting and two different pools of the CBDL samples were blotted. Quantification of NLRP3 was normalized to total protein load. Data are presented as mean ± SD. * *p* < 0.05, ** *p* < 0.01, *** *p* < 0.001.

Since previous reports described the involvement of apoptosis in cholestatic liver damage and as CBDL was associated with induction of the pro-apoptotic ER stress marker CHOP in our study, we evaluated the expression of caspase 3, executor of both the intrinsic and extrinsic apoptotic pathway, ER stress-induced caspase 12, and tumor necrosis factor receptor superfamily member 1a (TNFRsf1a) and Fas-Associated protein with Death Domain (FADD), two markers involved in extrinsic apoptotic cell death. Caspase 3 mRNA expression was significantly upregulated starting at week 1 (with the exception of weeks 2 and 3), and caspase 12 was significantly increased at all timepoints after CBDL, compared to sham surgery (Figure 2a). TNFRsf1a and FADD were upregulated at all timepoints in CBDL mice compared to their matching control group (Figure 2a). Besides apoptosis, the possible involvement of inflammasome activation, which results in pyroptotic cell death, was assessed by measuring caspase 1 and nucleotide-binding, leucine-rich repeat and pyrin domains-containing protein 3 (NLRP3) inflammasome levels. Although caspase 1 gene expression was increased from week 3 until week 6 in CBDL mice (Figure 2b), no increase in NLRP3 protein expression was demonstrated (Figure 2c).

### 2.3. Treatment with TUDCA Reduces the Degree of Liver Fibrosis

**Figure 3:**
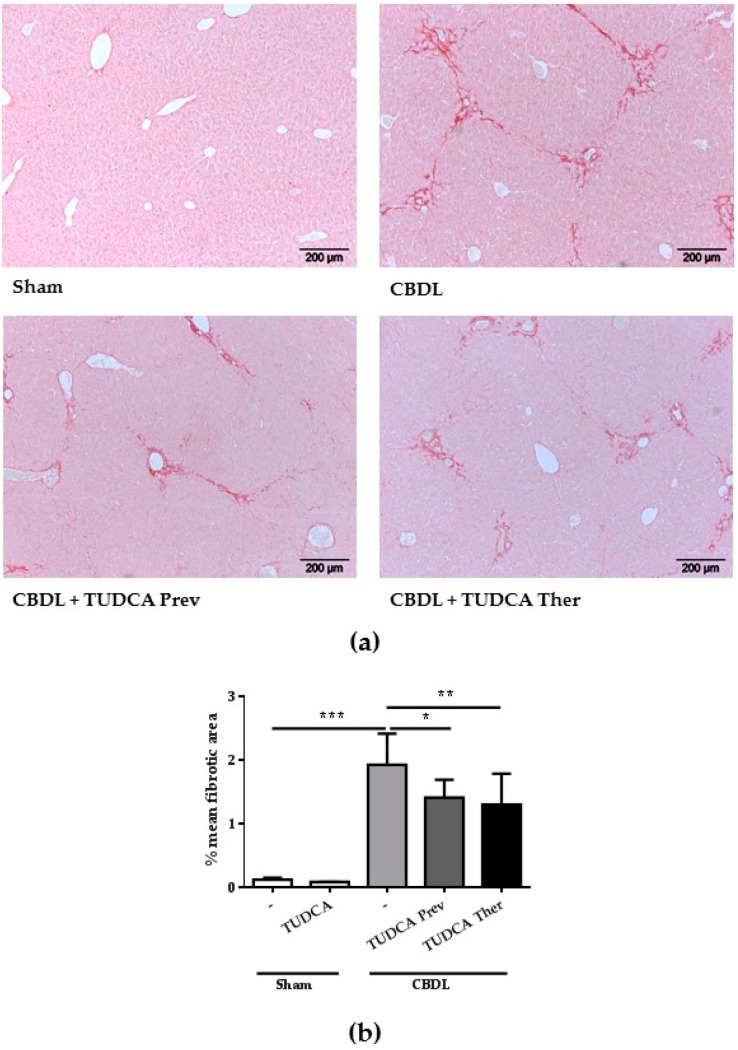
Treatment with TUDCA reduces the degree of liver fibrosis: (**a**) Representative histological images of livers from a sham mouse, a CBDL control and CBDL mice treated with TUDCA, stained with Sirius red. Original magnification 100×; (**b**) Computerized quantification of fibrosis scores (mean fibrotic area (%) ± SD). * *p* < 0.05, ** *p* < 0.01, *** *p* < 0.001.

As we demonstrated the induction of ER stress and involvement of apoptotic cell death in this animal model of cholestatic liver disease, we next investigated the therapeutic potential of TUDCA, which is known for its ER stress-reducing and anti-apoptotic capacities, on the degree of CBDL-induced liver fibrosis. In a preventive and a therapeutic study, TUDCA was administered starting one week before and two weeks after surgical induction until the end of the experiments respectively. As expected, the CBDL procedure resulted in a significant increase in fibrotic area compared to sham surgery (Figure 3). Importantly, TUDCA treatment, both in the preventive and the therapeutic setting, led to a significant reduction of liver fibrosis (Figure 3). This was associated with reduced, however not significantly reduced, liver damage, as reflected by AST and ALT levels (Table 1).

### 2.4. Treatment with TUDCA Reduces CBDL-Induced CHOP and Pro-Apoptotic Markers

**Figure 4:**
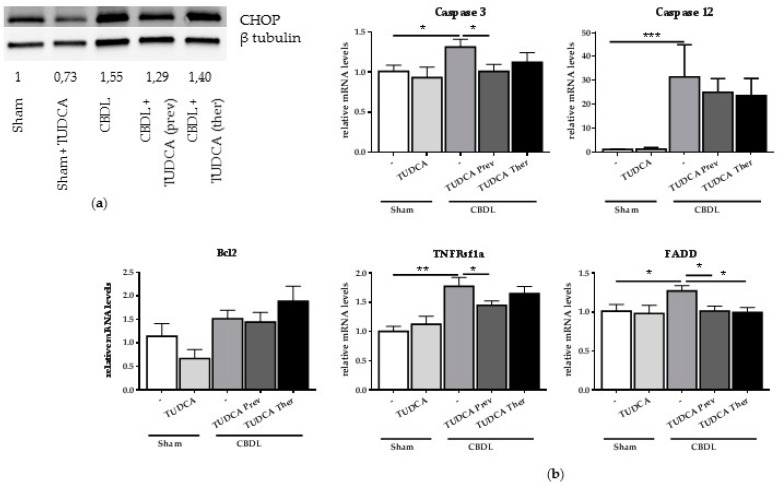

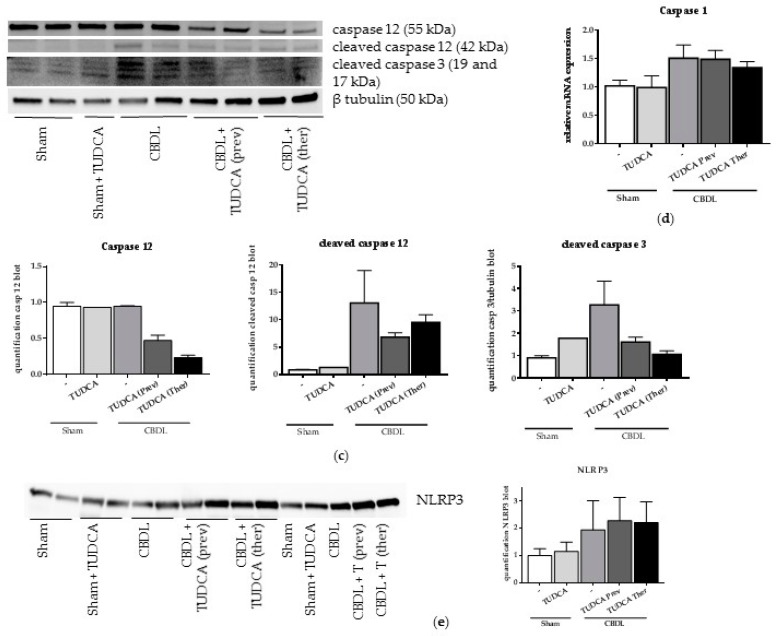
Treatment with TUDCA reduces CBDL-induced CHOP and pro-apoptotic markers. (**a**) Representative Western blot of CHOP on whole liver lysates. β-Tubulin was used for normalization. Quantification of CHOP blot compared to β-tubulin bands. Samples of three to five mice per group were mixed for blotting; (**b**) Real-time qPCR analysis of pro-apoptotic genes caspase 3 and 12, TNFRSf1a, FADD and Bcl2. Data are presented as mean ± SD. * *p* < 0.05, ** *p* < 0.01, *** *p* < 0.001; (**c**) Western blot and quantification of cleaved caspase 3 and cleaved caspase 12 on whole liver lysates. β-Tubulin was used for normalization. One to two samples per group were loaded with one sample consisting of a mixture of two mice; (**d**) Real-time qPCR analysis of pro-pyroptotic marker caspase 1. Data are presented as mean ± SD; (**e**) Western blot of NLRP3 on whole liver lysates. Quantification of NLRP3 was normalized to total protein load. Three samples per group were loaded with one sample consisting of a mixture of two mice, so a total of six mice were analyzed.

In order to explore whether TUDCA exerts its beneficial effect on liver damage and fibrosis through a reduction in pro-apoptotic UPR signaling, UPR-induced CHOP expression and gene and protein expression of different pro-apoptotic markers after TUDCA treatment was studied. Western blot analysis confirmed the induction of the pro-apoptotic transcription factor CHOP at 6 weeks after CBDL and demonstrated reduced CHOP expression when TUDCA was given preventively (Figure 4a). Preventive supplementation with TUDCA also significantly reduced mRNA levels of caspase 3 and both preventive and therapeutic TUDCA treatment reduced cleaved caspase 3 and cleaved caspase 12 protein levels, compared to the CBDL controls (Figure 4b,c). TNFRsf1 expression was only decreased in the preventive setting whereas FADD was significantly lower following the preventive and therapeutic TUDCA treatments. Although an increased trend was observed in the CBDL and therapeutic TUDCA group, the anti-apoptotic Bcl2, was not significantly altered after the CBDL or TUDCA treatment (Figure 4b). No effect of TUDCA, neither preventively nor therapeutically, was seen on caspase 1 gene expression in CBDL mice nor on NLRP3 levels (Figure 4d,e).

## 3. Discussion

### 3.1. CBDL Induces Hepatic Unfolded Protein and Pro-Apoptotic Response

Cholestatic liver disease is a major cause of liver fibrosis, which is triggered by hepatic cell death. It has been shown in different in vitro models that toxic bile acids, such as deoxycholic acid and glycochenodeoxycholic acid, cause ER stress and activation of the UPR [6,7,8,30]. In later studies, UPR activation has been investigated in different in vivo mouse models for cholestasis, which led to conflicting results [9,12,31,32]. However, none of these studies explored the UPR at different stages during early onset and progression of cholestatic liver disease. Therefore, we investigated the kinetics of hepatic UPR activation and the mode of associated hepatic cell death in a mouse model of cholestasis-induced liver fibrosis. In accordance with Mencin et al. and Tamaki et al. [12,31], we observed no increase in the UPR markers XBP1u and XBP1s (IRE1 pathway) and peIF2α and ATF4 (PERK pathway). GADD34 was increased starting from week 3 at the mRNA level, however, no significant differences in protein levels could be detected. Interestingly, we demonstrated gradually increasing levels of CHOP during the progression of liver fibrosis, which is one of the main UPR downstream effectors able to induce hepatic apoptotic and/or pyroptotic cell death when overexpressed [14]. Indeed, in case of excessive ER stress, CHOP promotes apoptosis through reduced expression of the anti-apoptotic protein Bcl-2, with subsequent Ca^2+^ influx into the cytoplasm [33]. Through m-Calpain, pro-caspase 12 is activated by cleavage, which further drives the caspase cascade [33]. Sequential activation of caspases plays a central role in the execution phase of apoptosis, in which caspase 3 is the final common executor of both the intrinsic and extrinsic apoptotic pathway. In the intrinsic pathway, mitochondrial permeabilization causes cytochrome c release, which activates caspase 9 and in turn caspase 3. In the extrinsic pathway, caspase 3 is activated by caspase 8 which is upstream bound and activated by FADD, which is bound to death receptors such as TNFR, forming the death-inducing signaling complex. In the present study, we demonstrate increased (cleaved) caspase 3 and 12, FADD and TNFRsf1a expression in fibrotic mice, indicative for the occurrence of apoptotic cell death during the initiation and progression of biliary liver damage and the development of fibrosis [34,35,36,37]. In addition, activation of CHOP is known to stimulate transcription of pro-caspase 1 and 11, which become activated upon cleavage and activated by NLRP3, the most important inflammasome [14]. Active caspase 1 and 11 in turn lead to apoptosis, through caspase 3, but also to pyroptosis, a programmed form of cell death, characterized by DNA damage, cell lysis and active IL-1β and IL-18 release [14]. Recently, the combination of CHOP-induced apoptosis and NLRP3 inflammasome-mediated pyroptosis has been demonstrated to be involved in the pathogenesis of experimental non-alcoholic steatohepatitis, which was successfully counteracted by treatment with TUDCA [14]. In our study, increased gene expression of caspase 1 in CBDL mice could be demonstrated, without, however, any change in NLRP3 protein levels, suggesting the absence of NLRP3 inflammasome-mediated pyroptosis. Summarized, these findings point towards a central role of CHOP-induced pro-apoptotic pathways in liver disease due to biliary etiology.

### 3.2. Treatment with TUDCA Reduces Liver Damage and Liver Fibrosis through Modulation of CHOP and Its Pro-Apoptotic Response

The chemical chaperone TUDCA, the hydrophilic taurine-conjugated form of UDCA, is known for its cytoprotective capacities of reducing ER stress and apoptotic cell death [15,20,29,30,38]. Since we demonstrated that CHOP-induced pro-apoptotic pathways are involved in cholestatic liver damage and fibrosis, and as TUDCA is known to be able to modulate these pathways in other diseases [25,27,39], we investigated its therapeutic potential to impede liver fibrosis in the CBDL mouse model. Importantly, both preventive and therapeutic treatment with TUDCA led to a significant reduction in the degree of liver fibrosis, which was accompanied by an improving trend for ALT and AST levels. These findings are in line with previous research which demonstrated that CHOP knockout mice develop less fibrosis [12]. A recent small double-blind randomized trial evaluated the therapeutic efficacy of TUDCA (750 mg daily) in liver cirrhosis, using UDCA as a parallel control, with a follow-up period of six months. They concluded that TUDCA therapy is safe and appears to be more effective than UDCA in improving biochemical parameters [40]. Our study evaluated the effect of TUDCA in a long term cholestasis mouse model, whereas other studies often study the effect during early cholestasis [41,42].

Lastly, we suggest that TUDCA indeed exerts its beneficial effect on liver damage and fibrosis through alleviating CHOP-induced apoptotic cell death, as treatment with TUDCA led to decreased CHOP expression and reduced levels of the pro-apoptotic markers caspase 3 and 12, TNFRsf1a and FADD.

In conclusion, this study indicates that CHOP-mediated apoptotic cell death contributes to the development of cholestasis-induced liver fibrosis and that treatment with TUDCA has a beneficial effect on liver fibrosis in this model of biliary fibrosis, which might, at least in part, be the result of its ER stress-reducing and anti-apoptotic properties. In this way, TUDCA might represent a therapeutic strategy for the treatment of biliary liver disorders.

## 4. Materials and Methods

### 4.1. Animals

Male 7-week-old Swiss and Sv129 mice, purchased from Harlan (Horst, The Netherlands) and Iffa Credo (Brussels, Belgium), were housed in the animal facility of the Faculty of Medicine and Health Sciences, Ghent University, Ghent, Belgium, and acclimatized under controlled conditions for one week prior to the experiments. They received care in accordance with Federation for Laboratory Animal Science Associations (FELASA) guidelines and the national guidelines for animal protection. The animal protocols used in this work were evaluated and approved by the Ethical Committee of Experimental Animals at the Faculty of Medicine and Health Sciences, Ghent University, Belgium, (ECD 13/12 approved on 29 April 2013 and ECD 14/67 approved on 3 November 2014).

### 4.2. Induction of Secondary Biliary Fibrosis

Secondary biliary fibrosis was induced by common bile duct ligation (CBDL) at the age of eight weeks, as described previously [29]. In brief, under isoflurane (Isoflo^®^, Abbott, Louvain-la-Neuve, Belgium) inhalation anaesthesia, a midline abdominal incision was made and the common bile duct was isolated and ligated with two knots of a non-resorbable suture (Silkan^®^ 5/0, Braun Aesculap, Tuttlingen, Germany). The first ligature was made below the junction of the hepatic ducts and the second was made above the entrance of the pancreatic duct. The common bile duct was resected between the two ligatures after which the abdomen was closed by suturing the abdominal muscle and skin in two separate layers. Control mice were sham-operated; the common bile duct was isolated but not ligated.

### 4.3. Kinetics of Hepatic UPR and Cell Death in Cholestasis-Induced Liver Fibrosis

In order to study the hepatic UPR and cell death during the time course of biliary fibrosis following CBDL, male Swiss mice were euthanized on a weekly basis, for six consecutive weeks, starting at week 1 after surgical induction (6 sham and 6 CBDL groups, 7–14 animals in each group). At sacrifice, liver samples were immediately harvested into liquid nitrogen and kept at −80 °C for protein and mRNA extraction. A portion was fixed in 4% formaldehyde for 24 h and imbedded in paraffin for histological analysis.

### 4.4. Preventive and Therapeutic Study with Tauroursodeoxycholic Acid

The therapeutic potential of TUDCA in CBDL mice was studied in both a preventive and therapeutic study. For the preventive study, male Sv129 CBDL and sham mice received 10 mg/kg/day TUDCA (Calbiochem, Overijse, Belgium) in their drinking water, starting one week before surgical induction till the moment of euthanasia. In the therapeutic setting, TUDCA (10 mg/kg/day) was added to the drinking water, starting 14 days after surgical induction till euthanasia. Control CBDL and sham mice received normal drinking water. This makes a total of five groups, a sham group with TUDCA, a sham group without TUDCA, a CBDL control group without TUDCA treatment, a preventive CBDL group and a therapeutic CBDL group (6–10 animals/group, Table 1). Six weeks after sham or CBDL surgery, retro-orbital blood was taken under ketamine (100 mg/kg, Ceva santé, Brussels, Belgium) and xylazin (10 mg/kg, Kela, Sint-Niklaas, Belgium) anesthesia after which the animals were euthanized by cervical dislocation. Liver samples were collected as described above.

### 4.5. Evaluation of Liver Fibrosis

The degree of liver fibrosis was scored quantitatively with Cell^D software (version 3.4, Olympus, Berchem, Belgium) on Sirius red-stained liver sections. A minimum of ten fields per liver section per mouse were blindly evaluated and scored by two independent researchers (AP and SR).

### 4.6. qPCR

RNA was extracted from 20 mg of frozen liver tissue preserved in RNA-later (Ambion, Thermo Fisher, Ghent, Belgium), according to the manufacturer’s guidelines (Rneasy Mini Kit, Qiagen, Venlo, The Netherlands) and measured for purity and quantity with spectrophotometry (Nanodrop, Thermo Scientific, Waltham, MA, USA). cDNA was made out of one microgram of mRNA by reverse transcription using the iScript cDNA synthesis kit (BioRad, Temse, Belgium) according to the manufacturer’s instructions. Diluted cDNA was subjected to 45 cycles of quantitative PCR amplification using SYBR Green mix (Sensimix, Bioline Reagents Ltd., London, UK) and 2 µM of each primer. A two-step program was run on a LightCyclerR 480 (Roche, Vilvoorde, Belgium). Melting curve analysis confirmed primer specificities. All reactions were run in duplicate and normalized to reference genes that showed stable expression in all samples (GAPDH, SDHA, HMBS and HPRT). The double Δ*C*q method was used for processing of qPCR data in which data were compared to the PBS control group. The PCR-efficiency of each primer pair was calculated using a standard curve of reference cDNA. Amplification efficiency was determined using the formula 10^−1/slope^ − 1. The primer set sequences are listed in Table S1.

### 4.7. Western Blotting

Total protein extract was obtained by homogenizing tissue in RIPA buffer (PBS, 0.5% NP-40, 0.5% sodium deoxycholate, 0.1% sodium dodecyl sulfate, 5.5% β-glycerophosphate, 1 mM dithiothreitol and complete protease and phosphatase inhibitors (Roche Diagnostics, Vilvoorde, Belgium)). The total protein yield was determined using Bradford reagent (Biorad, Temse, Belgium) and 30 µg protein was fractionated by SDS-PAGE. Protein lysates from multiple mice per group were pooled and transferred to a nitrocellulose membrane which was subsequently blocked for 1 h with 5% BSA, incubated overnight at 4 °C with primary antibodies in blocking buffer (Table S2), followed by 1 h incubation at room temperature with horse radish peroxidase-conjugated secondary antibodies (goat-anti rabbit sc2004, Santa Cruz, Heidelberg, Germany or goat anti-mouse sc2005, Santa Cruz). Clarity western ECL substrate (Biorad, Temse, Belgium) was used to visualize the proteins. GAPDH and β-tubulin were used as loading control proteins or total protein load was used for normalization.

### 4.8. Statistical Analysis

Data analysis was performed using GraphPad (GraphPad Software Inc., San Diego, CA, USA) and SPSS 21 (IMB Corp, Armonk, NY, USA). Differences between groups were calculated using *t*-test (for two groups) or one-way ANOVA analysis with Sidak’s correction (for multiple groups) for normally distributed data. For non-parametric data, multiple groups were compared using the Kruskal-Wallis test with Dunn’s post hoc test. Reported *p*-values were two-sided and considered significant when lower than 0.05.

## 5. Conclusions

Our data indicate that TUDCA has a beneficial effect on liver fibrosis in a mouse model of cholestatic liver disease, and suggest that this effect might, at least in part, be attributed to decreased hepatic CHOP and pro-apoptotic signaling.

## Figures and Tables

**Table 1:** ALT and AST levels.

Group	Number of Animals	ALT	AST
sham	6	24.05 ± 5.60	101.20 ± 36.17
sham + TUDCA	7	27.23 ± 8.36	96.43 ± 36.84
CBDL	9	312.3 ± 85.26 ***	505.3 ± 124 ***
CBDL + TUDCA preventive	10	274.4 ± 68.75	480.0 ± 121.8
CBDL + TUDCA therapeutic	8	246.3 ± 52.54 ^*p* = 0.095^	413.4 ± 94.49

Data are represented as mean ± SD. *** *p* < 0.001: CBDL vs. sham. ALT = alanine aminotransferase; AST = aspartate aminotransferase; TUDCA = tauroursodeoxycholic acid.

## Supplementary Materials

Supplementary materials can be found at www.mdpi.com/1422-0067/18/1/214/s1.
