# Supplementary material for: Modulation of the Unfolded Protein Response by Tauroursodeoxycholic Acid Counteracts Apoptotic Cell Death and Fibrosis in a Mouse Model for Secondary Biliary Liver Fibrosis
Source: Int J Mol Sci. 2017 Jan 20;18(1):214. doi: 10.3390/ijms18010214 (PMC5297843; doi:10.3390/ijms18010214)
Supplement: Supplementary file 1 [file ijms-18-00214-s001.pdf]

# Supplementary Materials: Modulation of the Unfolded Protein Response by Tauroursodeoxycholic Acid Counteracts Apoptotic Cell Death and Fibrosis in a Mouse Model for Secondary Biliary Liver Fibrosis

**Annelies Paridaens, Sarah Raevens, Lindsey Devisscher, Eliene Bogaerts, Xavier Verhelst, Anne Hoorens, Hans van Vlierberghe, Leo A. van Grunsven, Anja Geerts and Isabelle Colle**

**Table S1.** Primers used for quantitative polymerase chain reaction (qPCR) experiments. The PCR-efficiency of each primer pair was calculated using a standard curve of reference cDNA. Amplification efficiency was determined using the formula $(10^{-1/\text{slope}} - 1) \times 100$.

| Gene | Forward Primer 5′–3′ | Reverse Primer 3′–5′ | Efficiency | $R^2$ |
|---|---|---|---|---|
| *Gapdh* | CATGGCCTTCCGTGTTCCTA | GCGGCACGTCAGATCCA | 87.5 | 0.99 |
| *Hmbs* | AAGGGCTTTTCTGAGGCACC | AGTTGCCCATCTTTCATCACTG | 100.9 | 0.99 |
| *Hprt* | GTTAAGCAGTACAGCCCCAAA | AGGGCATATCCAACAACAAACTT | 74.8 | 0.98 |
| *Sdha* | CTTGAATGAGGCTGACTGTG | ATCACATAAGCTGGTCCTGT | 94.3 | 0.99 |
| *Caspase 1* | AATACAACCACTCGTACACGTC | AGCTCCAACCCTCGGAGAAA | 99 | 0.99 |
| *Caspase 3* | GCACTGGAATGTCATCTCGCT | GGCCCATGAATGTCTCTCTGAG | 105 | 0.99 |
| *Caspase 12* | AGACAGAGTTAATGCAGTTTGCT | TTCACCCCACAGATTCCTTCC | 98 | 0.98 |
| *Fadd* | TGTTCTCCCCAAACACACAA | CTGATGGGAGGGATTTCTGA | 115 | 0.99 |
| *Il1b* | CAACCAACAAGTGATATTCTCCATG | GATCCACACTCTCCAGCTGCA | 105 | 0.99 |
| *Tnfa* | CATCTTCTCAAAATTCGAGTGACAA | TGGGAGTAGACAAGGTACAACCC | 92.7 | 0.99 |
| *Tnfrsf1a* | CCACGCACTGGAAGTGTGT | CGGTGTGTGGCTGTAAGGAG | 92 | 0.99 |

**Table S2.** Antibodies used for Western blotting experiments. The specificity, isotype, catalog number and dilution factor of the antibodies are indicated.

| Antigen | Antibody Isotype, Clone | Company | Cat No. | Dilution |
|---|---|---|---|---|
| ATF4 | Rabbit polyclonal IgG | Santa Cruz | sc-200 | 1/500 |
| CHOP | Mouse monoclonal IgG2a, L63F7 | Cell Signaling | 2895 | 1/1000 |
| eIF2α | Rabbit polyclonal IgG | Cell Signaling | 9721 | 1/1000 |
| P-eIF2α | Rabbit monoclonal IgG, 119A11 | Cell Signaling | 3597 | 1/1000 |
| GADD34 | Rabbit polyclonal IgG, H193 | Santa Cruz | sc-8327 | 1/500 |
| Cleaved Caspase 3 | Rabbit polyclonal IgG | Cell Signaling | 9661 | 1/1000 |
| Caspase 12 | Rabbit polyclonal IgG | Cell Signaling | 2202 | 1/1000 |
| β-tubulin | Rabbit polyclonal IgG | Abcam | ab6046 | 1/500 |
| GAPDH | Rabbit polyclonal IgG | Abcam | ab9485 | 1/2500 |
| NLRP3 | Mouse Monoclonal IgG2b | Adipogen | AG-20B-0014-C100 | 1/3000 |
